# Linking Clinical Blood Metabogram and Gut Microbiota

**DOI:** 10.3390/metabo13101095

**Published:** 2023-10-19

**Authors:** Petr G. Lokhov, Elena E. Balashova, Dmitry L. Maslov, Oxana P. Trifonova, Andrey V. Lisitsa, Yulia M. Markova, Valentina V. Stetsenko, Anna S. Polyanina, Svetlana A. Sheveleva, Khaider K. Sharafetdinov, Dmitry B. Nikityuk, Victor A. Tutelyan, Alexander I. Archakov

**Affiliations:** 1 Institute of Biomedical Chemistry, 10 Building 8, Pogodinskaya Street, 119121 Moscow, Russiadlmaslov@mail.ru (D.L.M.);; 2 Federal State Budgetary Institution of Science, Federal Research Centre of Nutrition, Biotechnology and Food Safety, Ustinsky Passage 2/14, 109240 Moscow, Russia; yulia.markova.ion@gmail.com (Y.M.M.);

**Keywords:** metabogram, gut microbiota, gut microbiome, metabolomics, blood, diagnostics, mass spectrometry, clinical blood test, personalized metabolomics, clinical metabolomics

## Abstract

Recently, a clinical blood metabogram was developed as a fast, low-cost and reproducible test that allows the implementation of metabolomics in clinical practice. The components of the metabogram are functionally related groups of blood metabolites associated with humoral regulation, the metabolism of lipids, carbohydrates and amines, lipid intake into the organism, and liver function, thereby providing clinically relevant information. It is known that the gut microbiota affects the blood metabolome, and the components of the blood metabolome may affect the composition of the gut microbiota. Therefore, before using the metabogram in the clinic, the link between the metabogram components and the level of gut microorganisms should be established. For this purpose, the metabogram and microbiota data were obtained in this work for the same individuals. Metabograms of blood plasma were obtained by direct mass spectrometry of blood plasma, and the gut microbiome was determined by a culture-based method and real-time polymerase chain reaction (PCR). This study involved healthy volunteers and individuals with varying degrees of deviation in body weight (*n* = 44). A correlation analysis determined which metabogram components are linked to which gut microorganisms and the strength of this link. Moreover, diagnostic parameters (sensitivity, specificity and accuracy) confirmed the capacity of metabogram components to be used for diagnosing gut microbiota alterations. Therefore, the obtained results allow the use of the metabogram in a clinical setting, taking into account its relationship with gut microbiota.

## 1. Introduction

The advent of clinical metabogram is a response to the challenge of introducing metabolomics into clinical practice [1,2]. The metabolome is a global biochemical profile of biological objects, which reflects all the biochemical processes occurring in the body and which dynamically changes under the influence of both internal and external factors. The metabolome of blood, which is a connecting medium for all cells of the body, can justifiably be called the “molecular phenotype” of the whole organism, reflecting all the processes taking place in the body, whether it be features inherent in genes that are realized through translation and then transcription in enzymes, the products of which are metabolites [3,4].

Despite the fact that metabolomics, which measures the sets of metabolites that form the metabolome, has existed for more than two decades, and the methods used in it are already perfect in many respects; however, its use in medicine is extremely limited. One of the reasons for this is precisely the perfection of measurements—the accurate measurement of many metabolites at a single run [5], resulting in the complexity of metabolomic measurements, which is the reason for their high cost, duration of execution, complexity of data processing, problems of standardization and reproducibility of measurements [6,7].

Such characteristics are not consistent with clinical tests, where the speed of execution, low cost, reproducibility and interpretation of results accessible to physicians are the obvious requirements [7]. The metabogram is a compromise solution—a simplified way to analyze the blood metabolome with the execution parameters of clinical tests [8]. This is achieved by replacing the identification and analysis of individual metabolites by processing groups of metabolites. This is based on the assumption that covariant metabolites have similar, or at least unidirectional, causes and therefore, to a certain extent, can be evaluated jointly.

**Figure 1:**
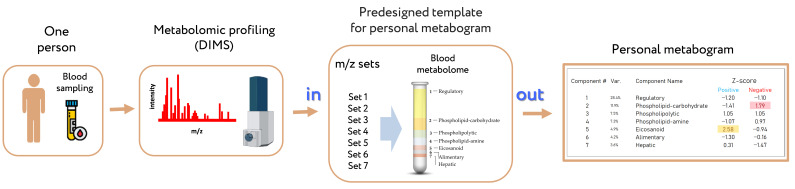
Workflow for obtaining a personal blood metabogram. Sampled blood, after sample preparation in order to separate the metabolome fraction, is subjected to direct-infusion mass spectrometry (DIMS). The resulting mass peaks are aligned with the characterized sets of mass spectrometric peaks corresponding to the components of the metabogram (predesigned template of personal metabogram), and their intensities are converted into Z-score scales and averaged over each group to obtain metabogram components showing the state (normal, upregulated or downregulated) of the blood metabolome (i.e., clinically relevant information). Adapted from [8].

This assumption was confirmed in previous work [8]. The seven main covarying groups of blood metabolites that are used to form the metabogram components describe 70 percent of blood metabolome variance and have compositional specificity and biological meaning. The composition of groups was established by determining its enrichment with metabolite classes. In addition, clinical tests were used as monitors of the state of the body to explain the biological meaning of these groups. Now, to quickly assess up to 70 percent of the variance in the blood metabolome, it is enough to take sets of mass spectrometric peaks assigned to these groups, convert their intensities to a Z-score, average them in each group and thus obtain a state (normal, upregulated or downregulated) of blood metabolome components (Figure 1) [8].

The clinical significance of this approach was demonstrated in previous work involving volunteers with metabolic disorders of varying severity associated with various degrees of obesity [9]. However, the blood metabolome is affected by many factors, among them the genome, diet and gut microbiota [10]. Moreover, blood metabolites may also affect some of these factors, e.g., it is suggested that the gut microbiota composition is affected by steroids [11,12,13,14]. Therefore, to implement a clinical blood metabogram, it is necessary to link the components of the metabogram with these factors so the interpretation of the metabogram is more scientifically justified. In this work, the linkage between the components of the metabogram and the gut microbiota was studied.

## 2. Materials and Methods

### 2.1. Subjects

Healthy, underweight, overweight and obese volunteers (total *n* = 44) were examined by the Clinic of Medicinal Nutrition at the Federal Research Centre of Nutrition, Biotechnology and Food Safety (Moscow, Russia). The groups of cases included volunteers with obesity of varying stages with a diagnosis of E 66.0, according to the International Classification of Diseases (obesity of exchange-alimentary origin). Subject selection, blood sampling, mass spectrometry analysis and gut microbiota analysis were conducted within the frame of a previous metabolomics study conducted in 2020 [15] and supported by the Program of the Presidium of the Russian Academy of Sciences (“Proteomic and Metabolomic Profile of Healthy Human”).

### 2.2. Mass Spectrometry Analysis of Blood Samples

Venous blood sampling, sample preparation, mass spectrometry analysis, mass spectra processing were conducted as described previously on the same equipment (maXis hybrid quadrupole time-of-flight mass spectrometer with an electrospray ionization source) and with the same materials [15]. Standardization of mass peak intensities was performed by dividing the intensity by the standardization value, which was calculated for each peak separately as follows: a 50 Da range (which started at 25 Da before and ended at 25 Da after the *m*/*z* of the mass peak) was selected; all peaks inside the range were sorted in a descending order according to their intensities; the intensity of the 150th peak was selected as the standardization value. Standardized intensities improved the further use of correlation analysis due to the correction of ion suppression of peak intensities [15]. Standardized mass lists were normalized by applying the *normalize* function (which brings the sum of the intensities of the peaks in the spectrum to 1) of the Matlab program (version R2019a, MathWorks, Natick, MA, USA). Alignment of the *m*/*z* values of the mass peaks between different mass spectra was performed as described previously [16]. The alignment algorithm used was previously specially developed and tested for the high-resolution mass spectra of blood metabolites obtained by direct-infusion mass spectrometry (DIMS) and implemented as an iterative process based on the detection of correlation of mass spectrometry peak patterns.

### 2.3. Design of Metabogram (Template for Personal Metabograms)

**Figure 2:**
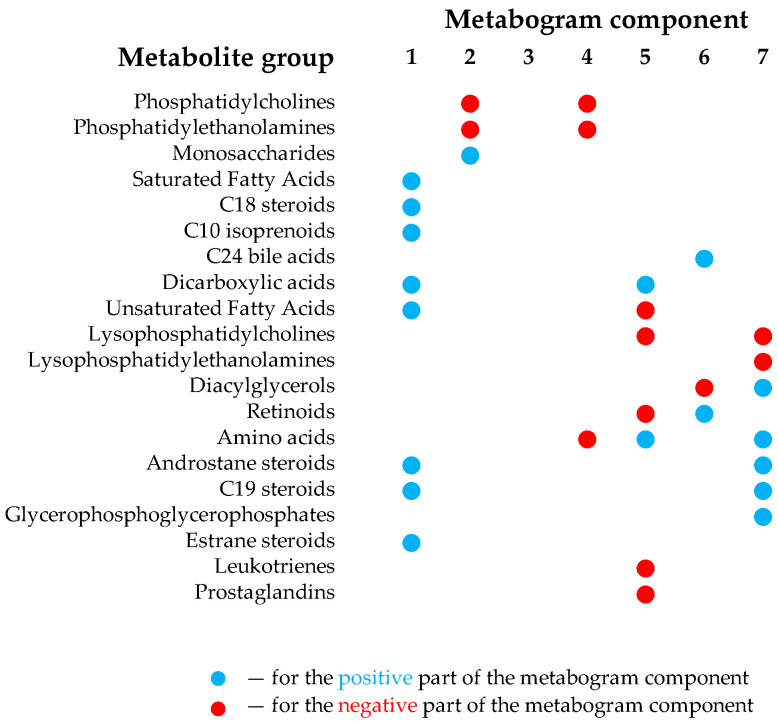
Composition of the clinical blood metabogram components. Adapted from [8].

The design of the metabogram using a reference cohort of healthy subjects was conducted in previous work, and the details of this are described in [8]. Briefly, to design the metabogram, blood plasma samples of 48 healthy subjects (reference cohort) were analyzed using DIMS (Figure 1). After data preprocessing (alignment, standardization and normalization), the resulting lists of mass peaks were analyzed using principal component analysis (PCA). The sets of mass peaks corresponding to the highest positive or lowest negative principal component coefficients (loadings) formed the blood metabolome components (BMCs). The sets of mass peaks for the first seven BMCs, explaining approximately 70% of blood metabolome variance, formed the metabogram components and were further used as template for quick-producing personal metabograms. Applying metabolite set enrichment analysis (MSEA) [17], the composition of metabogram components was determined by identifying the chemical classes with which they are enriched (Figure 2). To clarify the biological specificity of the metabogram components, clinical blood tests (*n* = 71) were used. Due to the fact that the principal components have positive and negative coefficients (loadings) involved in the formation of the metabogram components, each metabogram component has two Z-score scales reflecting their measure, called “positive” and “negative”, respectively. The Z-score is a common way of representing data on a unitless scale and is the raw score minus the population mean, divided by the population standard deviation. With a normal distribution, the Z-score is connected to the *p*-values; for example, 1.64 corresponds to *p* = 0.05 (one-tailed), which is thought to be the cutoff for statistical significance and enables the detection of the sample’s deviation from the population. Z-scores of the metabogram components in the −1.64 to +1.64 range are in the normal range; up- and downregulation correspond to higher and lower Z-score values, respectively.

The components of the metabogram are functionally related groups of the blood metabolites associated with humoral regulation (component 1 called “regulatory”), lipid–carbohydrate metabolism (component 2), phospholypolysis (component 3 called “phospholipolytic”), lipid–amine metabolism (component 4), the level of different metabolites including oxidized fatty acids (component 5 called “eicosanoid”), lipid intake into the organism (component 6 called “alimentary”) and liver function (component 7 called “hepatic”), thereby providing clinically relevant information.

### 2.4. Personal Metabograms

**Figure 3:**
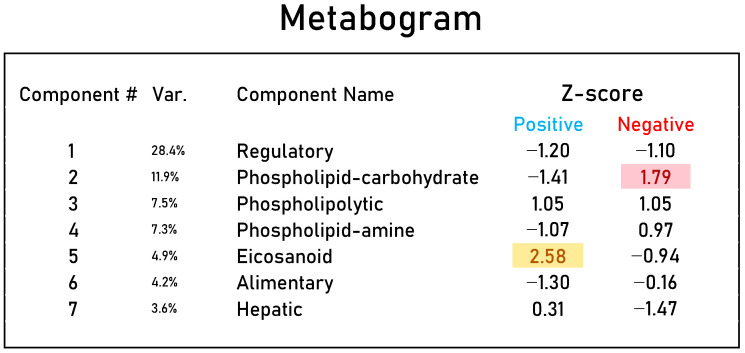
An example of a clinical blood metabogram. The Z-score value is a measure of the metabogram component (from −1.64 to +1.64 is the normal range). “Up-” and “downregulation” of the blood metabolic groups related to metabogram components correspond to higher and lower Z-scores, respectively. The deviated components of the metabogram are selected by background color: red indicates upregulation in the corresponding metabogram component; yellow indicates downregulation in the corresponding metabogram component. “Var” column shows the percentage of the variance explained by the metabogram component. Adapted from [9].

Personal metabograms, which are in fact the prototype of the result of a clinical laboratory test, were obtained using the study cohort (see Section 2.1), which consisted of subjects with normal, overweight and obese bodies. The mass lists were standardized, normalized and then aligned with the *m*/*z* values of the metabogram template (i.e., with seven *m*/*z* sets corresponding to seven metabogram components) developed using the reference cohort (see Section 2.3). Then, the Z-scores for the metabogram components, reflecting the increase or decrease in the concentration of metabolites comprising them, were calculated using the mass peak intensities (by averaging the Z-scores for peaks belonging to the same metabogram component) [8]. Figure 3 shows an example of a metabogram.

### 2.5. Gut Microbiota Analysis

Sampling, transportation and storage of the test material were carried out in accordance with the methodological recommendations “Taking, transporting, storing clinical material for PCR diagnostics” developed by the “Central Research Institute of Epidemiology” of the Federal Service for Surveillance on Consumer Rights Protection and Human Wellbeing (Moscow, 2012). For the study, samples of the feces after natural defecation were used. The study is carried out before the start of taking antimicrobial drugs and immunomodulators or 12–14 days after the end of taking the drugs. Exclusion criteria: women during menstruation; patients who took 3–4 days before the study laxatives, castor or Vaseline oil, used an enema or rectal suppositories. For 1–3 days before sampling, patients followed a diet that excluded foods that enhance fermentation processes in the intestines, lactic acid products, alcohol and also excluded the use of antibiotics and bacterial preparations (containing bifidobacteria, lactobacilli, *E. coli*, etc.). Fecal samples were taken into a sterile plastic container. The container with the material was delivered to the laboratory and stored at +2–8 °C until the start of the study. The time from taking the material to the start of the culture-based study did not exceed 6 h. After taking a part of the sample for culture analysis, the samples were frozen at −20 °C for further real-time polymerase chain reaction (PCR) analysis.

#### 2.5.1. Gut Microbiota Analysis by Culture-Based Method

Gut microbiota was determined by inoculation of selective culture media. The inoculation algorithm with appropriate dilutions and incubation times is summarized in Table 1. One gram of fecal sample was homogenized in 9 mL of thioglycol–phosphate buffer (composition: KH_2_PO_4_—4.5 g/L (Chimmed, Moscow, Russia), Na_2_HPO_4_·2H_2_O_4_—15.1 g/L (Chimmed, Russia), agar—1 g/L (FBSI SSC PMB, Obolensk, Russia), thioglycolic acid—0.4 mL (Merck, Rahway, NJ, USA), 1 n NaOH was used for pH adjustment to pH 6.8). Subsequent tenfold serial dilutions were made in 9 mL of thioglycol–phosphate buffer. For detection of bifidobacteria and sulfite-reducing clostridia, 1 mL of the required dilutions (Table 1) were inoculated in the tubes with semiliquid media, for detection of other groups of microorganisms, 50 μL of dilutions were inoculated on the surface of the agar differential-diagnostic culture media with subsequent spreading with a sterile spatula. All media were prepared following the manufacturer’s instructions. Just before the start of the work, the thioglycol–phosphate buffer and semiliquid media were heated on a boiling water bath for 15 min and cooled to 40–45 °C to reduce the dissolved oxygen content. For the determination of anaerobic microorganisms, bifidobacteria, sulfite-reducing clostridia, lactic acid bacteria and bacteroides, incubation was carried out under anaerobic conditions using gas-generating bags for chemical oxygen binding “AnaeroGen™” (Oxoid). The antagonistic (acid-forming) activity of bifidobacteria was assessed by determining the pH of the culture fluid (corn–lactose medium) on the 5th day of incubation using a pH meter. The criteria were pH limits: less than 4.5—antagonistically active bifidobacteria; 4.6–5.1—weak antagonism; more than 5.1—absence of antagonistic activity.

#### 2.5.2. Gut Microbiota Analysis by Real-Time PCR

Besides the cell-based method, the gut microbiota was determined through the gut microbiome (collection of intestinal microbial genes) analysis. Gut microbiome was determined by using a kit “Colonoflor-16” (Alfalab LLC, St. Petersburg, Russia). The kit is intended for quantitative assessment of the state of microbiocenosis of the large intestine in children and adults by polymerase chain reaction with fluorescent detection of amplification results in real time. The studied material is fecal samples. The kit makes it possible to detect the DNA of obligate representatives of the microbiota of the large intestine as well as opportunistic microorganisms including:

Normal flora and anaerobic microorganisms:

- Total bacteria
- *Lactobacillus* spp.
- *Bifidobacterium* spp.
- *Faecalibacterium prausnitzii*
- *Bacteroides thetaiotaomicron*
- *Bacteroides* spp./*Faecalibacterium prausnitzii* ratio

Opportunistic microorganisms that cause inflammation, diarrhea and dyspepsia:

- *Klebsiella pneumoniae*
- *Klebsiella oxytoca*
- *Enterobacter* spp. and *Citrobacter* spp.
- *Clostridium difficile*
- *Clostridium perfringens*
- *Staphylococcus aureus*
- *Proteus vulgaris* and *Proteus mirabilis*
- *Candida* spp. yeast

Pathogenic microorganisms, causative agents of acute intestinal infections:

- *Escherichia coli enteropathogenic*
- *Salmonella* spp.
- *Shigella* spp.

Microorganisms as markers of disease (their detection or change in the number can signal various pathological conditions):

- *Fusobacterium nucleatum*
- *Parvimonas micra*

DNA extraction from fecal samples was performed using the “AmpliSens^®^ DNA-sorb-C” kit (AmpliSens, Moscow, Russia) in accordance with the manufacturer’s instructions, with the additional use of a disintegrator (“1600 MiniG^®^” (SPEX SamplePrep, Metuchen, NJ, USA) and special tubes containing 100 µm quartz beads, 1.4 mm zirconia beads and 4 mm quartz beads (*2303-MM3*, SPEX SamplePrep). Real-time PCR amplification and detection were performed using “CFX96 Real Time System” (BIO-RAD, Hercules, CA, USA), the values of cycle quantification (Cq) were calculated automatically by the “CFX manager” software, the interpretation of the obtained results was performed using the “Colonoflor” software provided with the reagent kit.

### 2.6. Correlation Analysis

The link between metabogram components and the level of gut microorganisms was revealed by a correlation analysis. Spearman’s correlation between the Z-scores of the metabogram components showing their up- and downregulation and the gut microbiota test results for each person were calculated using the *corr* function of the Matlab program. The Cohen scale [18] identified a correlation of 0.5 as being on the cusp of “medium/moderate” (0.30–0.49) and “large/strong” (0.50–1.00). The function also returned the p-values for testing the hypothesis of no correlation against the alternative hypothesis of a nonzero correlation.

### 2.7. Plotting Links between Metabogram Components and Gut Microbiota

The correlation coefficients between the components of the metabogram and the level of microorganisms in the gut microbiota were used to construct a distance matrix using the *pdist* function of the Matlab program. Correlation was chosen as a distance measure. To project the components of the metabogram along with the components of the microbiota onto a two-dimensional plane, classical multidimensional scaling was used by applying the *cmdscale* function from Matlab. The eigenvalue *e* returned by the function allowed for an estimate of the minimum number of dimensions required for a correct reflection of the original data. The points on the plot corresponding to the components of the metabogram and microorganisms of the microbiota were connected by dashed lines, the thickness of which reflects the absolute value of the correlation between them. The color of the line codes the positive or negative value of this correlation. Lines were drawn for correlation coefficients >0.3 and <−0.3.

### 2.8. Diagnostic Parameters

To assess the diagnostic potential of the metabogram for diagnosing gut microbiota deviations from the norm, the following diagnostic parameters were evaluated: sensitivity—the percentage of correctly identified positive results (the deviation is correctly assigned to the metabogram component with a Z-score out of the threshold value); specificity—the percentage of correctly identified negative results (the deviation from the normal range is correctly not assigned to the metabogram component with a Z-score not exceeding the threshold value); and accuracy—the percentage of correctly identified positive and negative results.

The ROC curve was built by the *perfcurve* function (Matlab), which also returned sensitivity and specificity values for diagnostics depending on the selected threshold Z-score value and the optimal Z-score value for heist diagnostic accuracy.

## 3. Results

### 3.1. Studied Subjects

Forty-four volunteers—healthy, underweight, overweight and with obesity according to the World Health Organization’s classification of obesity by BMI—were examined by the medical board at the Federal State Budgetary Institution “Nutrition and Biotechnology” (Moscow, Russia). Table 2 summarizes the cohort characteristics.

### 3.2. Mass Spectrometry Data for Metabograms

**Figure 4:**
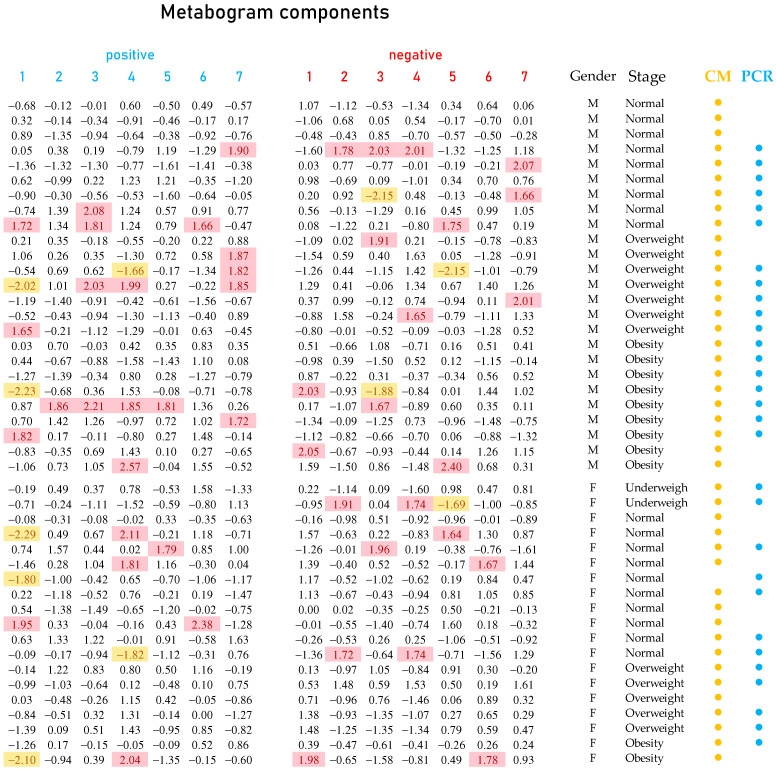
Metabogram data for subjects participating in the study. Each row with digital values corresponds to the Z-scores of the metabogram components for an individual (“positive” from 1 to 7 and “negative” from 1 to 7; see example in Figure 3), presented as a row. Z-score is a measure of the metabogram components (from −1.64 to +1.64 is the normal range; up- and downregulation correspond to higher and lower Z-score values, respectively). Background color coding: red indicates upregulation in the corresponding metabogram component; yellow indicates downregulation in the corresponding metabogram component. Labels: M, male; F, female; CM; culture-based method; PCR, real-time polymerase chain reaction method; (●) subjects whose gut microbiota was tested by culture-based method; (●) subjects whose gut microbiota was tested by real-time PCR.

Mass spectrometry of blood plasma generated typical mass spectra of the low-molecular-weight fraction of blood. Up to about *m*/*z* 600, peaks of metabolites of various classes and above *m*/*z* 600, intense peaks of various phospholipids were observed. On average, ~9.3 thousand peaks were detected in the spectrum. Aligned and standardized mass lists are presented in Table S1. These mass spectrometry data were used to obtain personal metabograms for all subjects participating in the study (Figure 4).

### 3.3. Metabogram Components Connection with Gut Microbiota Studied by Culture-Based Method

**Figure 5:**
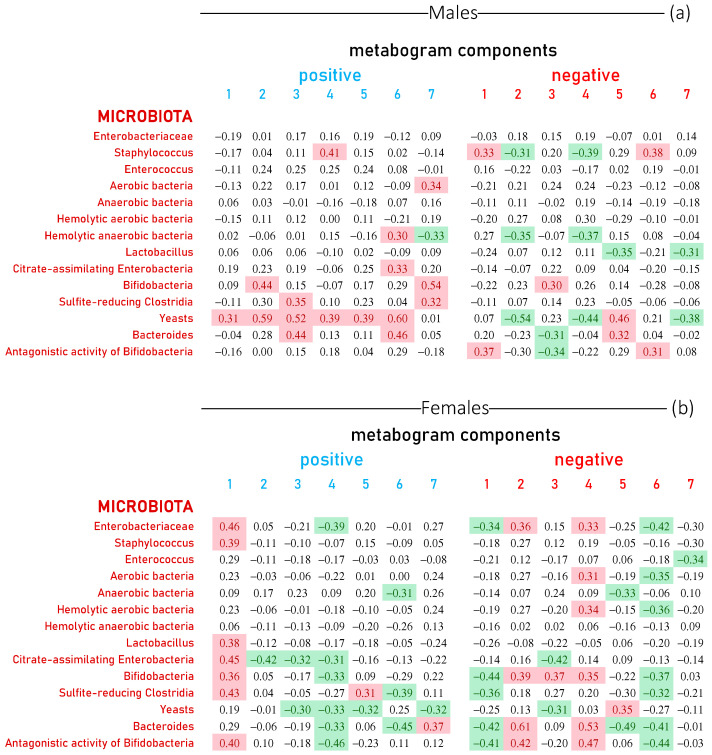
Correlation between the level of gut microorganisms and the metabogram components calculated for healthy individuals and individuals with various degrees of body weight deviation. Each row represents the correlation coefficients between the Z-scores of an individual’s metabogram components (“positive” from 1 to 7 and “negative” from 1 to 7; see Figure 3) and the results of his microbiota tests. The level of microorganisms in fecal samples was assessed by a culture-based method. Data are presented for males (**a**) and females (**b**). Correlation coefficients less than −0.3 and greater than 0.3 are highlighted in green and red backgrounds, respectively. The *p*-values for testing the hypothesis of no correlation against the alternative hypothesis of a nonzero correlation are presented in Figure S1.

In accordance with clinical practice for microbiota assessment, the amounts of both resident protective populations of the intestinal microbiota (lactobacilli, bifidobacteria, bacteroides, lactose-fermenting enterobacteria), opportunistic transient populations (hemolytic microorganisms, citrate-assimilating enterobacteria, staphylococci, yeasts and molds) and commensal clostridia and enterococci were evaluated in this study (Table S2). Statistical data for the gut microbiota test results calculated for males and females separately are presented in Table S3. The correlation of the metabogram components with gut microbiota assessed by a culture-based method is presented in Figure 5. In this figure, there are no data on coagulase positive staphylococci since this type of bacteria was not detected in the analysis (see Table S2).

In males, yeasts were the most correlated with metabogram components (10 out of 14 values show a positive or negative correlation). Next come bifidobacteria, bacteroides and staphylococci (Figure 5a). For women, the relationship between the components of the metabogram and the gut microbiota is more pronounced and systemic. Positive 1, negative 2 and 4 components generally positively correlate with all microorganisms, for many of which the correlation coefficient is >0.3. Furthermore, positive 4 and negative 1 and 6 components generally negatively correlate with all microorganisms, and some of them correlate with a correlation coefficient of <−0.3. Yeasts, bifidobacteria and bacteroides also correlate with metabogram components as for males. However, the correlation of enterobacteria and bacteroides with the components of the metabogram in women was more pronounced than in men (Figure 5b). Thus, an association of the blood metabolome, reflected in the metabogram, with the human gut microbiota assessed by a culture-based method was revealed, and this relationship is different for men and women.

### 3.4. Metabogram Components Connection with Gut Microbiota Studied by Real-Time PCR

**Figure 6:**
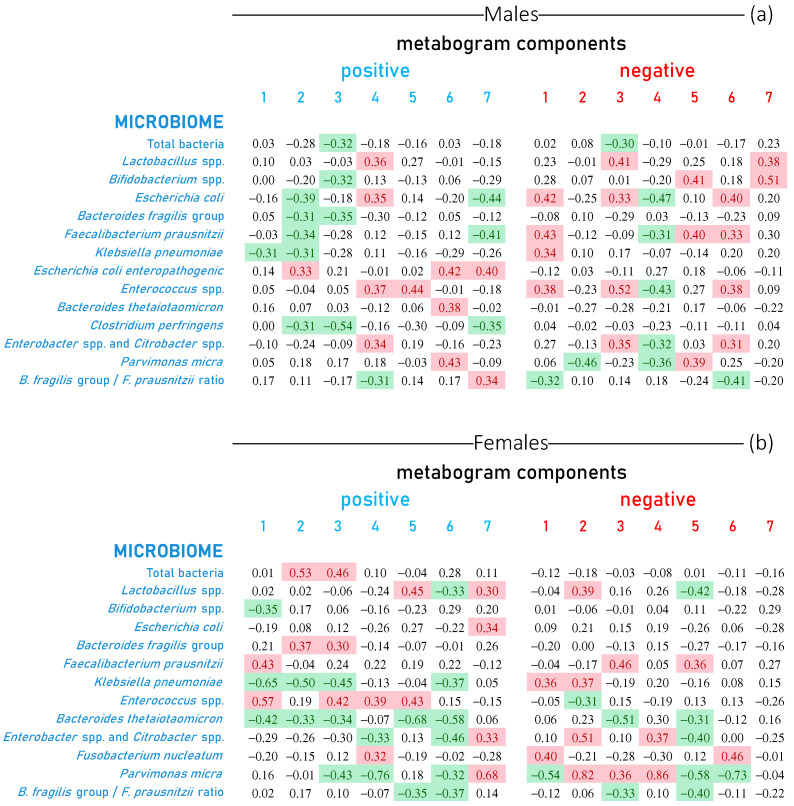
Correlation of gut microbiome with metabogram components calculated for healthy individuals and individuals with various degrees of body weight deviation. Each row represents the correlation coefficients between the Z-scores of an individual’s metabogram components (“positive” from 1 to 7 and “negative” from 1 to 7; see Figure 3) and the results of his microbiome tests. The level of microorganisms in fecal samples was assessed by real-time PCR analysis. Data are presented for males (**a**) and females (**b**). Correlation values less than −0.3 and greater than 0.3 are highlighted in green and red backgrounds, respectively. The p-values for testing the hypothesis of no correlation against the alternative hypothesis of a nonzero correlation are presented in Figure S2.

The correlation of the metabogram components with the gut microbiome (microbiota assessed by a real-time PCR analysis) is presented in Figure 6. In the figure, there are no data for *Klebsiella oxytoca*, *Staphylococcus aureus*, *Clostridium difficile*, *Proteus vulgaris/mirabilis*, *Candida* spp., *Salmonella* spp., *Shigella* spp., *Escherichia coli enteropathogenic* (for women) and *Clostridium perfringens* (for women) since these types of bacteria were not detected in the analysis or detected in a small number of samples (see Table S2).

Figure 6 shows that the relationship between the components of the metabogram and the gut microbiome is pronounced and different for men and women. It can be said that all components are associated with certain measured microorganisms. Considering that the components of the metabogram are ranked from 1 to 7 according to their coverage of blood metabolome variance, i.e., the first component is the weightiest, it can be said that in males, the relationship with *Klebsiella pneumoniae* is the most pronounced, followed by *Faecalibacterium prausnitzii*, *Escherichia coli* and *Enterococcus* spp.

In women, as well as in the microbiota measured by a culture-based method, the tendency remains in the microbiome measured by the real-time PCR: the association of the microorganism level with the components of the metabogram is more diverse and differs from that of men. Similar to men, there is also a pronounced link with *Klebsiella pneumoniae*, *Enterococcus* spp. and *Faecalibacterium prausnitzii*. However, a less prominent link with *Escherichia coli* and a stronger link with *Parvimonas micra* are detected.

### 3.5. Visualization of the Links between Metabogram Components and Gut Microbiota

**Figure 7:**
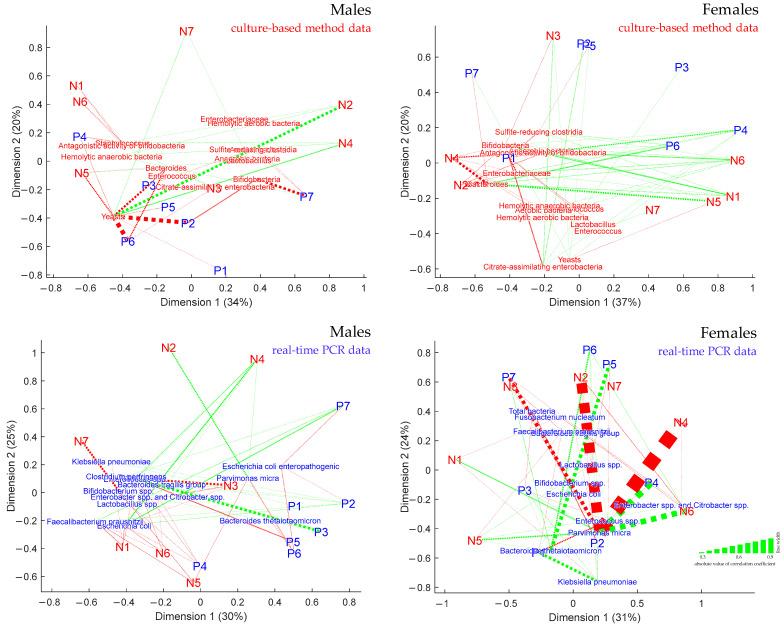
Linkage of clinical blood metabogram components with gut microbiota. Multidimensional scaling was used to build plots. Points labeled “**P**” and “**N**” correspond to positive and negative metabogram components, respectively. The names of microorganisms indicated in red and blue correspond to culture-based method and real-time PCR data, respectively. The linkage is shown by dashed lines. The width of the line corresponds to the strength of linkage (corresponds to the absolute value of the correlation coefficient), and the color of the line corresponds to a positive (red line) or negative correlation (green line). Lines are drawn for correlation coefficients >0.3 and <−0.3. The percentages of the eigenvalue *e* returned by the multidimensional scaling function are displayed in parenthesis.

The links between the components of the metabogram and the microorganisms in the gut microbiota, which is the main goal of this study, were represented by projecting the corresponding points onto a two-dimensional plane using classical multidimensional scaling. The eigenvalue *e* returned by the multidimensional scaling function validated the accuracy of such a projection (for the first two dimensions, *e* is more than 50%). As a result, Figure 7 accurately depicts on the plane the relationship between the components of the metabogram and the microorganisms of the microbiota. In addition, lines were used to represent the strength of the links, with the width of the lines reflecting the value of the correlation coefficient between the points.

### 3.6. Diagnostic Potential of Metabogram Components

**Figure 8:**
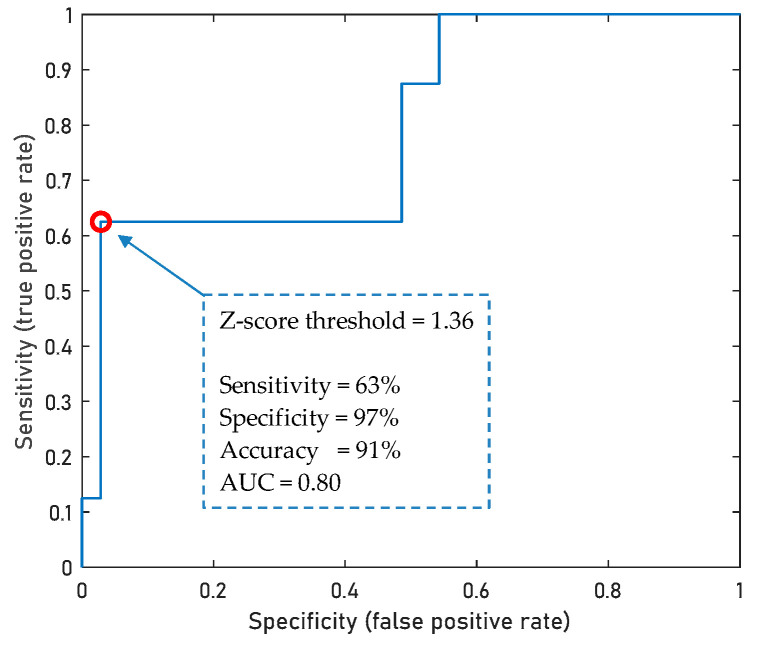
ROC curve for the diagnostics of yest levels exceeding the norm based on the Z-score value of positive component 6 of the metabogram. A total of 43 samples from cases (*n* = 8) and control individuals (*n* = 35) were used to build a ROC curve. The point shown on the ROC curve represents the maximum diagnostic accuracy value.

If the level of any microorganism in the gut microbiota is outside the normal range for several examined volunteers, then this allows us to evaluate the diagnostic capacity of the metabogram in relation to such a microorganism. In this study, yeasts measured by a culture-based method allowed this to be achieved. Figure 8 demonstrates that the Z-score value of the positive component 6 of the metabogram allows diagnosing yeast levels exceeding the norm (0–4 lg(CFU/g)) with an accuracy of 91%, sensitivity of 63% and specificity of 97%.

For the evaluation of diagnostic parameters for other microorganisms, an additional study is needed using a cohort aligned with the number of patients with gut microorganism levels outside the norm and patients with the normal level of the same microorganism.

## 4. Discussion

The challenges of implementing metabolomics in medicine [6,7] led to the concept of simplifying the N-of-1 metabolomics study that was realized in a blood metabogram. The metabogram approach makes it possible to abandon the identification of individual metabolites, of which there are thousands in biological samples, which makes any metabolomics study complex, time-consuming, laborious and expensive [8]. In the metabogram, only groups of related metabolites are processed, which composition is quickly assessed by the MSEA [17]. Thus, the complex identification of individual metabolites is replaced by a group analysis. Moreover, the group level evaluation leads to increased reproducibility of the data, so that the coefficient of variation (CV) [8] for the components of the metabogram is unattainable for most individual metabolites [19] and fully complies with the requirements to reproducibility for clinical laboratory tests.

The metabogram offers a variety of clinically relevant information from the blood metabolite groups involving regulation, lipid–carbohydrate and lipid–amine metabolism, eicosanoids, amino acids, lipid intake into the organism and liver function. The clinical value of the metabogram was already confirmed in a previous study by assessing metabolic alterations related to overweight and obesity [9]. However, before the metabogram is used in the clinic, it is necessary to define how the components of the metabogram relate to the gut microbiota due to its prominent impact on the blood metabolome [10]. For this, people with various metabolic changes associated with various degrees of body weight deviation were evaluated using the metabogram, and the obtained results were compared with their gut microbiota composition. Overweight and obese people are good models of metabolic alterations, which are well-described. The widespread presence of such people and the fact that imbalance in the gut microbiota may be a factor leading to obesity [20,21,22] justified the chosen cohort for linking the metabogram with the gut microbiota.

Up to 100 trillion microorganisms live in the gut, called the gut microbiota, which is unique to each person and contains tens of times more cells and 100 times more genes than the human body’s own genes [23,24]. The diversity of the gut microbiota requires a choice of microorganisms to link to the metabogram components. Since the metabogram is a way of using metabolomics in clinical practice, it was worthwhile to investigate the relationship with microorganisms routinely measured in clinics. It is these microorganisms that were measured in this work by the culture-based method and using the “Colonoflor-16” kit for the real-time PCR analysis, which is widely used in Russian clinics.

The results of this study showed a deep relationship between the metabogram components and the gut microbiota, which fully corresponds to the scientific data available today. It was observed that gender-specific differences in the gut microbiota are associated with metabogram component values, in particular with the first metabogram component, which is enriched with steroids (Figure 2).

Differences in the composition of the microbiota depending on sex have also been established in numerous studies. Thus, newborn boys have a greater number of *Bifidobacterium* (*Actinobacteria* type) and *Clostridiales bacteria* (*Firmicutes* type) and a lower number of *Enterobacteriales* bacteria (*Proteobacteria* type) [11,12]. At the same time, girls have a greater diversity of gut microbiota compared to boys. This points to a possible role of steroid hormones in shaping the composition of the microbiota. According to studies performed in adults, young and middle-aged women have a greater diversity of gut microbiota than men, and after 40 years, these differences cease to be significant [13,14]. A significant strong direct relationship has been demonstrated between the estrogen levels and the taxonomic diversity of bacteria within the Clostridia class. A South Korean study of 57 people demonstrated a relationship between the levels of sex hormones such as testosterone and estradiol and the composition of the gut microbiota [25]. Thus, at present, significant gender differences in the composition of the gut microbiota associated with sex hormones have been identified, which are consistent with the sex-specific linkage of the metabogram components with the gut microbiota, especially component 1 due to the enrichment of this component with steroid hormones (Figure 2).

The effect of gut microorganisms on the blood metabolome, due to the fact that a significant part of the metabolites in the blood comes from the intestine, is also well known and consistent with the results obtained. Thus, the total bacteria (changes in nutrition or age-related changes, intestinal disorders), *Lactobacillus* spp. (imbalanced diet, food allergy), *Bacteroides fragilis* and *Bacteroides fragilis*/*Faecalibacterium prausnitzii* ratio (dysbiotic disturbances, intestinal inflammation), yeasts (systemic mycoses, non-invasive mycotic process, specific dysbiosis, including by disorders of carbohydrate metabolism), etc., correlated with the components of the metabogram.

Therefore, the goal of this study was achieved; it was confirmed that the components of the metabogram are associated with the gut microorganisms. It was established which components of the metabogram are most strongly associated with which types of microorganisms. Therefore, when interpreting metabograms for their use in the clinic, this information can be taken into account. It should also be noted that the blood metabogram, as a new method for analyzing metabolomic data, gave an updated version of the relationship between the gut microbiota and the human molecular phenotype expressed in the blood metabolome. In particular,

- The difference between the sexes was clearly shown.
- A precise metric of the blood metabolome/gut microbiota relationship was provided (the portion of the metabolome covered by each component of the metabogram is indicated in the metabogram, and the strength of connection is expressed by the correlation coefficient).
- New data about blood metabolome/gut microorganism relations were revealed (e.g., the strong connection of the metabolome with the yeast levels).
- A high diagnostic capacity of blood metabolites (by means of a metabogram) in relation to gut microbiota was demonstrated.

Moreover, almost all deviations in the measured microorganisms are associated with either malnutrition and/or intestinal pathologies. Therefore, the established connection between the components of the metabogram and gut microorganisms can be considered not only in the interpretation of the metabogram but also in the diagnosis of intestinal diseases.

The outcomes of this research can also be assessed in light of fundamental knowledge. The metabogram’s capacity to quantify the relationship between the blood metabolome and microbiota revealed a strong linkage between them. This fact enables us to argue that the blood metabolome, a global molecular biochemical phenotype, is the result of a superposition of the body’s internal factors and gut microbiota composition. This finding supports theories that attribute a significant role to the microbiota in how the human body functions. The gut microbiota is even credited with the ability to change human behavior, including eating habits [26,27,28], and, as a result, is associated with the metabolic features of the organism and the risks of the most common diseases [29,30,31]. The established significant link between the microbiota and the components of the metabogram, formed by regulatory (steroids, eicosanoids) and nutritive (phospholipids, carbohydrates, amino acids) substances, provides reliable proof for this. Given the expanded significance of the microbiota, this fact places additional demands on the study and interpretation of the blood metabolome as well as the evaluation of human health.

The limitations of the work include the fact that all known types of bacteria that affect the blood metabolome [32,33], including in the obesity, were not investigated [20,34,35]. However, this was not the goal of this work and may in the future serve both as a more complete disclosure of the potential of the metabogram and as a subject of study of the influence of the microbiota on human health.

The prospect of further investigation of the metabogram lies in the study of the relationship of its components with the genome. It is expected that this will be another sign of the viability of the metabogram approach, and, as in the case of this work, it may provide new knowledge about blood metabolome relationships. Another prospect is a study of diagnostic parameters for a wide list of gut microorganisms and a more accurate assessment of diagnostic parameters at different values of the metabogram components. Such a study needs a cohort aligned in the number of patients with and without dysbiotic disturbances in the microbiota.

## 5. Conclusions

The metabolome is the level of organization of biological systems directly related to the global biochemical phenotype, the measurement of which for medical purposes is long-awaited and promising. The clinical metabogram is such an attempt, as it has the features of a clinical test in terms of performance and, as previously shown, carries clinically significant information. However, the blood metabolome is closely related to the human microbiota, and this relationship is shown in this work, which makes the interpretation of the metabograms more complete. Moreover, being an effective method for the analysis of blood, the metabogram allowed us to make a new global overview of the relationship of the blood metabolome with the microbiota, bringing a lot of new scientific information.

## Figures and Tables

**Table 1:** Conditions for gut microbiota analysis by culture-based method.

Groups of Microorganisms	Culture Media	Dilutions	Incubation
Enterobacteria	Endo agar (Biokompas—S, Moscow, Russia)	10^−3^, 10^−4^, 10^−5^, 10^−6^ 50 μL	37 °C, 24 h
Enterobacteria utilizing citrate	Simmons Citrate Agar (Biokompas—S, Russia)	10^−4^, 10^−5^ 50 μL	37 °C, 4 days
Bacteroides	Bacteroides Bile Esculin Agar with Bacteroides Selective Supplement (FD062) (HiMedia, Mumbai, India)	10^−4^, 10^−5^, 10^−6^, 10^−7^ 50 μL	37 °C, 48 h, anaerobic conditions
Total number of aerobic microorganisms, hemolytic microorganisms	Columbia Blood Agar (HiMedia) with 5% *v*/*v* sterile defibrinated sheep blood	10^−5^, 10^−6^ 50 μL	37 °C, 48 h
Total number of anaerobic microorganisms, hemolytic anaerobes	Columbia Blood Agar (HiMedia) with 7% *v*/*v* sterile defibrinated sheep blood	10^−6^, 10^−8^ 50 μL	37 °C up to 7 days
Lactic acid bacteria	MRS agar with sorbic acid additive (Biokompas—S)	10^−4^, 10^−5^, 10^−6^, 10^−7^ 50 μL	37 °C, 3 days
Enterococci	Kanamycin esculin azide agar (105222) (Merck)	10^−4^, 10^−6^ 50 μL	37 °C, 48 h
Staphylococci	Baird-Parker Agar with egg yolk and tellurite additive (Biokompas—S)	10^−3^, 10^−5^ 50 μL	37 °C, 48 h
Bifidobacteria	Corn–lactose medium (Biokompas—S)	10^−7^, 10^−8^, 10^−9^, 10^−10^ 1 mL	37 °C, 5 days anaerobic conditions
Sulfite-reducing clostridia	Iron–sulfite medium (Biokompas—S)	10^−6^, 10^−7^, 10^−8^, 10^−9^ 1 mL	37 °C, 5 days anaerobic conditions
Yeasts and molds	Sabouraud agar (Biokompas—S) with streptomycin	10^−1^, 10^−2^, 10^−3^ 50 μL	30°C, 5 days

**Table 2:** Study cohort characteristics.

Subjects	Age (Years)	Body Mass Index (kg/m^2^)	Gender (Number)
Cohort for culture-based method testing of gut microbiota			
Males (Normal—9, Overweight—7, Obesity—9)	Males 30.4 ± 6.8 ^1^	Males 27.7 ± 5.3	Males—25
Females (Underweight—2, Normal—9, Overweight—5, Obesity—2)	Females 30.3 ± 5.2	Females 24.9 ± 7.1	Females—18
Cohort for real-time PCR testing of gut microbiota			
Males (Normal—6, Overweight—5, Obesity—7)	Males 30.9 ± 7.0	Males 27.7 ± 4.4	Males—18
Females (Underweight—2, Normal—5, Overweight—4, Obesity—1)	Females 31.6 ± 5.0	Females 24.4 ± 6.5	Females—12

^1^ mean ± standard deviation.

## Data Availability

The data presented in this study are available as Supplementary Materials and on request from the corresponding author.

## Supplementary Materials

The following supporting information can be downloaded at https://www.mdpi.com/article/10.3390/metabo13101095/s1. Table S1: Aligned and standardized mass lists. Table S2: Gut microbiota test results. Table S3: Statistical data for gut microbiota test results calculated for males and females separately. Figure S1: *p*-Values for the correlation of gut microorganism levels (assessed by a culture-based method) with the metabogram components calculated for healthy individuals and individuals with various degrees of body weight deviation. Figure S2: *p*-Values for the correlation of gut microorganism levels (assessed by a real-time PCR analysis) with the metabogram components calculated for healthy individuals and individuals with various degrees of body weight deviation.
