# Supplementary material for: Linking Clinical Blood Metabogram and Gut Microbiota
Source: Metabolites. 2023 Oct 19;13(10):1095. doi: 10.3390/metabo13101095 (PMC10609303; doi:10.3390/metabo13101095)
Supplement: Supplementary file 1 [file metabolites-13-01095-s001.zip › Figure S1, Figure S2, Table S3.pdf]

Supplementary Materials

Males (a)

MICROBIOTA	positive 1	positive 2	positive 3	positive 4	positive 5	positive 6	positive 7	negative 1	negative 2	negative 3	negative 4	negative 5	negative 6	negative 7
Enterobacteriaceae	0.36	0.96	0.40	0.44	0.35	0.57	0.67	0.87	0.39	0.47	0.35	0.72	0.98	0.49
Staphylococcus	0.40	0.85	0.60	0.04	0.47	0.93	0.51	0.09	0.13	0.32	0.05	0.15	0.06	0.67
Enterococcus	0.58	0.24	0.21	0.21	0.24	0.69	0.95	0.42	0.28	0.88	0.41	0.93	0.35	0.97
Aerobic bacteria	0.52	0.28	0.41	0.96	0.56	0.65	0.09	0.31	0.31	0.24	0.25	0.26	0.55	0.68
Anaerobic bacteria	0.79	0.88	0.96	0.42	0.38	0.73	0.43	0.58	0.60	0.91	0.36	0.51	0.35	0.39
Hemolytic aerobic bacteria	0.46	0.60	0.55	0.99	0.59	0.30	0.36	0.33	0.18	0.69	0.14	0.16	0.62	0.95
Hemolytic anaerobic bacteria	0.92	0.77	0.97	0.46	0.44	0.14	0.10	0.18	0.08	0.75	0.07	0.45	0.69	0.83
Lactobacillus	0.77	0.78	0.77	0.63	0.92	0.65	0.66	0.24	0.75	0.55	0.59	0.08	0.30	0.13
Citrate-assimilating Enterobacteria	0.36	0.27	0.35	0.75	0.21	0.10	0.32	0.48	0.74	0.27	0.67	0.85	0.33	0.47
Bifidobacteria	0.65	0.02	0.47	0.74	0.41	0.16	0.00	0.29	0.25	0.13	0.19	0.51	0.17	0.69
Sulfite-reducing Clostridia	0.60	0.14	0.08	0.62	0.26	0.87	0.11	0.60	0.74	0.50	0.26	0.81	0.76	0.76
Yeasts	0.12	0.002	0.01	0.05	0.05	0.001	0.95	0.72	0.004	0.25	0.03	0.02	0.30	0.06
Bacteroides	0.84	0.17	0.02	0.54	0.60	0.02	0.80	0.33	0.26	0.13	0.84	0.11	0.86	0.92
Antagonistic activity of Bifidobacteria	0.44	0.99	0.46	0.38	0.86	0.15	0.37	0.06	0.14	0.09	0.28	0.16	0.12	0.71

Females (b)

MICROBIOTA	positive 1	positive 2	positive 3	positive 4	positive 5	positive 6	positive 7	negative 1	negative 2	negative 3	negative 4	negative 5	negative 6	negative 7
Enterobacteriaceae	0.06	0.85	0.41	0.11	0.42	0.95	0.28	0.17	0.14	0.55	0.18	0.32	0.08	0.23
Staphylococcus	0.11	0.66	0.69	0.77	0.54	0.73	0.85	0.47	0.27	0.63	0.46	0.86	0.53	0.23
Enterococcus	0.25	0.67	0.47	0.50	0.91	0.89	0.74	0.41	0.63	0.51	0.78	0.82	0.48	0.17
Aerobic bacteria	0.35	0.89	0.82	0.38	0.98	0.99	0.35	0.47	0.27	0.53	0.22	0.44	0.16	0.46
Anaerobic bacteria	0.73	0.50	0.36	0.73	0.43	0.22	0.30	0.57	0.78	0.34	0.72	0.19	0.82	0.69
Hemolytic aerobic bacteria	0.36	0.80	0.95	0.48	0.69	0.84	0.34	0.44	0.29	0.44	0.16	0.56	0.14	0.42
Hemolytic anaerobic bacteria	0.83	0.67	0.61	0.72	0.43	0.30	0.62	0.52	0.94	0.94	0.82	0.53	0.60	0.72
Lactobacillus	0.13	0.64	0.75	0.51	0.48	0.85	0.34	0.30	0.74	0.39	0.83	0.80	0.42	0.46
Citrate-assimilating Enterobacteria	0.06	0.08	0.20	0.22	0.52	0.60	0.37	0.57	0.52	0.08	0.57	0.71	0.59	0.57
Bifidobacteria	0.14	0.83	0.50	0.19	0.73	0.24	0.38	0.07	0.11	0.13	0.16	0.38	0.13	0.92
Sulfite-reducing Clostridia	0.08	0.89	0.83	0.28	0.21	0.11	0.67	0.14	0.47	0.28	0.44	0.23	0.19	0.41
Yeasts	0.45	0.95	0.23	0.18	0.20	0.31	0.20	0.31	0.61	0.20	0.91	0.16	0.28	0.66
Bacteroides	0.24	0.82	0.45	0.18	0.82	0.06	0.14	0.09	0.01	0.73	0.02	0.04	0.09	0.97
Antagonistic activity of Bifidobacteria	0.10	0.70	0.47	0.06	0.37	0.67	0.63	0.09	0.08	0.42	0.05	0.81	0.07	0.91

Figure S1. p-Values for the correlation of gut microorganism levels (assessed by a culture-based method) with the metabogram components calculated for healthy individuals and individuals with various degrees of body weight deviation. The p-value tests the hypothesis of no correlation against the alternative hypothesis of a nonzero correlation. P-values <0.05 (rejection of the hypothesis that no correlation exists) are selected by the red color. Data is presented for males (**a**) and females (**b**).

Males (a)

MICROBIOME	metabogram components positive 1	2	3	4	5	6	7	negative 1	2	3	4	5	6	7
Total bacteria	0.91	0.26	0.20	0.48	0.53	0.91	0.48	0.92	0.74	0.22	0.69	0.96	0.51	0.37
Lactobacillus spp.	0.69	0.90	0.90	0.14	0.27	0.97	0.54	0.36	0.97	0.09	0.24	0.32	0.47	0.12
Bifidobacterium spp.	1.00	0.42	0.20	0.61	0.59	0.83	0.25	0.26	0.77	0.98	0.44	0.09	0.48	0.03
Escherichia coli	0.53	0.11	0.47	0.16	0.58	0.43	0.07	0.08	0.32	0.19	0.05	0.70	0.10	0.42
Bacteroides fragilis group	0.83	0.21	0.16	0.23	0.64	0.84	0.65	0.76	0.69	0.25	0.90	0.61	0.35	0.72
Faecalibacterium prausnitzii	0.92	0.16	0.26	0.64	0.56	0.62	0.09	0.08	0.64	0.72	0.22	0.10	0.18	0.23
Klebsiella pneumoniae	0.21	0.20	0.25	0.66	0.52	0.25	0.30	0.17	0.70	0.50	0.78	0.57	0.43	0.42
Escherichia coli enteropathogenic	0.58	0.18	0.39	0.98	0.94	0.08	0.10	0.63	0.90	0.65	0.28	0.47	0.81	0.66
Enterococcus spp.	0.86	0.88	0.83	0.13	0.07	0.97	0.47	0.12	0.35	0.03	0.07	0.28	0.12	0.72
Bacteroides thetaiotaomicron	0.54	0.79	0.90	0.64	0.81	0.12	0.94	0.96	0.28	0.26	0.39	0.49	0.83	0.37
Clostridium perfringens	0.99	0.21	0.02	0.53	0.23	0.73	0.15	0.88	0.95	0.91	0.36	0.68	0.67	0.89
Enterobacter spp. and *Citrobacter* spp.	0.70	0.34	0.72	0.17	0.44	0.52	0.36	0.27	0.61	0.15	0.20	0.89	0.22	0.43
Parvimonas micra	0.83	0.48	0.51	0.47	0.91	0.07	0.72	0.82	0.05	0.36	0.14	0.11	0.32	0.43
B. fragilis group / *F. prausnitzii* ratio	0.50	0.67	0.51	0.22	0.59	0.49	0.17	0.20	0.69	0.57	0.48	0.33	0.10	0.43

Females (b)

MICROBIOME	metabogram components positive 1	2	3	4	5	6	7	negative 1	2	3	4	5	6	7
Total bacteria	0.97	0.07	0.13	0.75	0.90	0.39	0.73	0.71	0.57	0.92	0.80	0.98	0.73	0.63
Lactobacillus spp.	0.95	0.95	0.85	0.46	0.14	0.30	0.34	0.91	0.22	0.62	0.42	0.18	0.57	0.37
Bifidobacterium spp.	0.26	0.59	0.86	0.61	0.48	0.37	0.53	0.97	0.85	0.97	0.90	0.74	0.50	0.37
Escherichia coli	0.55	0.80	0.70	0.42	0.40	0.49	0.28	0.77	0.50	0.63	0.55	0.41	0.86	0.37
Bacteroides fragilis group	0.51	0.24	0.34	0.66	0.83	0.97	0.42	0.54	0.99	0.68	0.64	0.40	0.59	0.63
Faecalibacterium prausnitzii	0.16	0.90	0.46	0.49	0.55	0.50	0.71	0.90	0.60	0.13	0.89	0.25	0.83	0.40
Klebsiella pneumoniae	0.02	0.10	0.14	0.68	0.90	0.23	0.89	0.25	0.23	0.54	0.53	0.63	0.81	0.65
Enterococcus spp.	0.05	0.56	0.17	0.21	0.16	0.65	0.64	0.89	0.33	0.64	0.55	0.68	0.69	0.41
Bacteroides thetaiotaomicron	0.17	0.29	0.28	0.82	0.01	0.05	0.84	0.85	0.48	0.09	0.34	0.32	0.72	0.62
Enterobacter spp. and *Citrobacter* spp.	0.36	0.41	0.35	0.29	0.68	0.13	0.30	0.76	0.09	0.75	0.24	0.20	0.99	0.43
Fusobacterium nucleatum	0.53	0.65	0.72	0.32	0.55	0.95	0.37	0.19	0.52	0.37	0.34	0.71	0.13	0.98
Parvimonas micra	0.62	0.97	0.16	0.004	0.57	0.31	0.01	0.07	0.001	0.26	0.0003	0.05	0.01	0.91
B. fragilis group / *F. prausnitzii* ratio	0.94	0.59	0.76	0.84	0.26	0.23	0.65	0.72	0.86	0.30	0.76	0.19	0.73	0.50

Figure S2. p-Values for the correlation of gut microorganism levels (assessed by a real-time PCR analysis) with the metabogram components calculated for healthy individuals and individuals with various degrees of body weight deviation. The p-value tests the hypothesis of no correlation against the alternative hypothesis of a nonzero correlation. P-values <0.05 (rejection of the hypothesis that no correlation exists) are selected by the red color. Data is presented for males (**a**) and females (**b**).

Table S3. Statistical data for gut microbiota test results calculated for males and females separately.

		Males		Females	
		mean	st.d.	mean	st.d.
**Culture-based method**	Enterobacteriaceae	6.97	1.08	7.30	1.01
	Staphylococcus	5.50	1.02	5.82	1.47
	Coagulase Positive Staphylococci	-	-	-	-
	Enterococcus	6.12	0.78	6.13	1.16
	Aerobic bacteria	7.62	0.82	7.86	0.84
	Anaerobic bacteria	10.04	0.84	9.44	0.98
	Hemolytic aerobic bacteria	7.25	0.86	7.42	1.35
	Hemolytic anaerobic bacteria	8.99	1.24	8.56	1.26
	Lactobacillus	7.20	0.92	7.21	1.11
	citrate-assimilating enterobacteria	6.22	0.93	5.92	0.71
	Bifidobacteria	8.68	0.85	8.61	1.09
	Sulfite-reducing Clostridia	7.16	0.99	7.22	0.88
	Yeasts	3.12	0.85	3.06	1.18
	Bacteroides	8.89	1.04	8.13	1.37
	Antagonistic activity of Bifidobacteria	4.30	0.22	4.17	0.16
**Real-time PCR analysis**	Total bacteria	13.55	0.62	13.83	0.40
	Lactobacillus spp.	8.75	1.11	8.73	1.10
	Bifidobacterium spp.	12.32	1.05	12.68	0.67
	Escherichia coli	9.43	0.99	10.07	0.68
	Bacteroides fragilis group	13.32	0.82	13.50	0.42
	Faecalibacterium prausnitzii	12.05	0.76	12.25	0.35
	Klebsiella pneumoniae	1.36	2.62	2.62	3.25
	Klebsiella oxytoca	0.38	1.60	1.05	2.46
	Candida spp.	-	-	-	-
	Staphylococcus aureus	-	-	-	-
	Escherichia coli enteropathogenic	2.93	3.46	-	-
	Enterococcus spp.	6.55	3.22	6.17	2.59
	Bacteroides thetaiotaomicron	10.05	2.78	6.55	5.05
	Clostridium difficile	-	-	-	-
	Clostridium perfringens	2.36	3.52	1.55	3.69
	Proteus vulgaris and *Proteus mirabilis*	-	-	-	-
	Enterobacter spp. and *Citrobacter* spp.	8.95	0.96	9.54	0.85
	Fusobacterium nucleatum	-	-	2.70	4.04
	Parvimonas micra	6.51	3.66	6.96	3.33
	Salmonella spp.	-	-	-	-
	Shigella spp.	-	-	-	-
	B. fragilis group / *F. prausnitzii* ratio	125.47	387.36	23.45	22.17
